# The British Journals: Toxicology

**Published:** 1839-01

**Authors:** 


### TOXICOLOGY.

*On the Poisonous Effects of the Substance used for what is termed Printing "in Gold."* By Gurney Turner, Esq. Surgeon.

[The following case is interesting and important. We are surprised that the exact composition of the poisonous powder has not yet been made known in any of our journals. The practice of "printing *in gold*" ought to be prevented by authority.]

A lad applied at the General Dispensary for relief of a most distressing itching of the scrotum. On examining the part, it seemed relaxed and inflamed, the sebaceous follicles considerably enlarged, and round the roots of the hairs were small scabs, caused by his scratching the part, to relieve the tingling sensation. The hair on the scrotum and pubes was of a decided grass-green colour, and though the irritation resembled that produced by the pediculus pubis, I could discover none of these vermin or their ova. On enquiry, the lad referred these symptoms to his occupation at a newspaper office, being engaged in printing the Golden Sun paper—so named from its golden type. It appears this hue is communicated by brushing a fine bronze-coloured powder (composed, according to the workmen's account, of copperas, verdigris, and quicksilver,) over the type, which is first printed in yellow ink. This powder is given to those employed in ounce packets, and about forty hands were thus employed, almost all of whom had been forced after a time to give up this work, some keeping at it only two days; others for a week or more; but all suffering more or less from its effects.

The hair on his head and in the axilla was of the same colour, and he complained of itching in these parts, and about the wrists, though in a less degree, and the hair felt peculiarly harsh, dry, and matted.

He stated that on the third day of being thus employed, he had been seized with vomiting of a green-coloured fluid, and a sensation of heat and constriction in the œsophagus, with pain in the stomach, which he referred to swallowing and inhaling portions of the powder diffused through the air of the room: this was followed by epistaxis, recurring at intervals, itching of the before-mentioned parts, more especially of the pubes and scrotum, tenderness of the epigastrium and bowels, accompanied by loss of appetite and rest.

He had ceased attending the dispensary after about nine or ten days, at which period he was quite well, only that the hair still continued green—but less intensely so.

This case having excited my curiosity, I asked and obtained permission to see the process of printing these papers. They are printed with a yellow ink, composed of size and gamboge, and then handed over to men who, with a common hat-brush, distribute the powder over the paper, which adheres to the moist printed portions. About a dozen persons were thus engaged when I visited the office, all of whom complained more or less of the same symptoms. Some added, that this irritating powder had caused deep ulcers on the genitals; others declared it had salivated them to a certain extent; but though their gums appeared slightly spongy, they hardly seemed more so than those of most persons whose stomachs are out of order; and I could not detect any mercurial fœtor.

I wished much to be allowed to have a portion of this powder for chemical examination, but my request could not be granted, as its composition is kept a secret. I was told it was prepared in Germany; it looked like very fine brass filings; the whole air of the room was loaded with it, and my coat glistened, as also did my face and hair, which rivalled in brightness the wig of Caligula, who had recourse to gold-dust to produce the effect I obtained so cheaply.

*Med. Gazette. November* 3, 1838.

